# Dysmegakaryopoiesis in myelodysplastic syndrome: Beyond cell dysplasia

**DOI:** 10.1016/j.htct.2024.09.2485

**Published:** 2025-02-15

**Authors:** Maria Mirele da Silva Ribeiro, Francisco Dário Rocha Filho, Howard Lopes Ribeiro Junior, Priscila da Silva Mendonça, Ronald Feitosa Pinheiro, Silvia Maria Meira Magalhães

**Affiliations:** a Laboratório de Citogenômica do Câncer, Centro de Pesquisa e Desenvolvimento de Medicamentos (NPDM), Universidade Federal do Ceará (UFC), Fortaleza, Ceará, Brazil; b Programa de Pós-Graduação em Ciências Médicas, Universidade Federal do Ceará (UFC), Fortaleza, Ceará, Brazil; c Hospital Universitário Walter Cantídio, Universidade Federal do Ceará (UFC), EBSERH, Fortaleza, Brazil

A 57‑year-old female was admitted with persistent normocytic anemia (hemoglobin: 9.6 g/dL; mean corpuscular volume: 94 fL; neutrophil count: 2.0 × 10^3^/L; platelet count: 199 × 10^9^/L). Causes of non-clonal cytopenias were all excluded: nutritional deficiencies, hormonal disorders, autoimmune and infectious diseases.

A bone marrow aspirate showed dyserythropoiesis and dysmegakaryopoiesis with the presence of micro-megakaryocytes. <1 % of blasts were identified, and an increase of medullar iron was present with no ringed sideroblasts. Immunohistochemical analysis with CD61 made micro-megakaryocytes highlighted dysplastic megakaryocytes in an unusual peritrabecular area (Figure 1). CD34 and p53 showed no abnormalities. Bone marrow G-banding cytogenetic analysis revealed 46,XX,del(11)(q23)[8]/46,XX[12], according to the International System for Human Cytogenomic Nomenclature. The diagnosis of myelodysplastic syndrome (MDS) with multilineage dysplasia was established, according to the World Health Organization (WHO) classification and risk-stratification. The Revised International Prognostic Scoring System (IPSS-R) score was very low. After seven years of follow-up, the patient is still alive and no disease progression has been observed.

**Figure 1:**
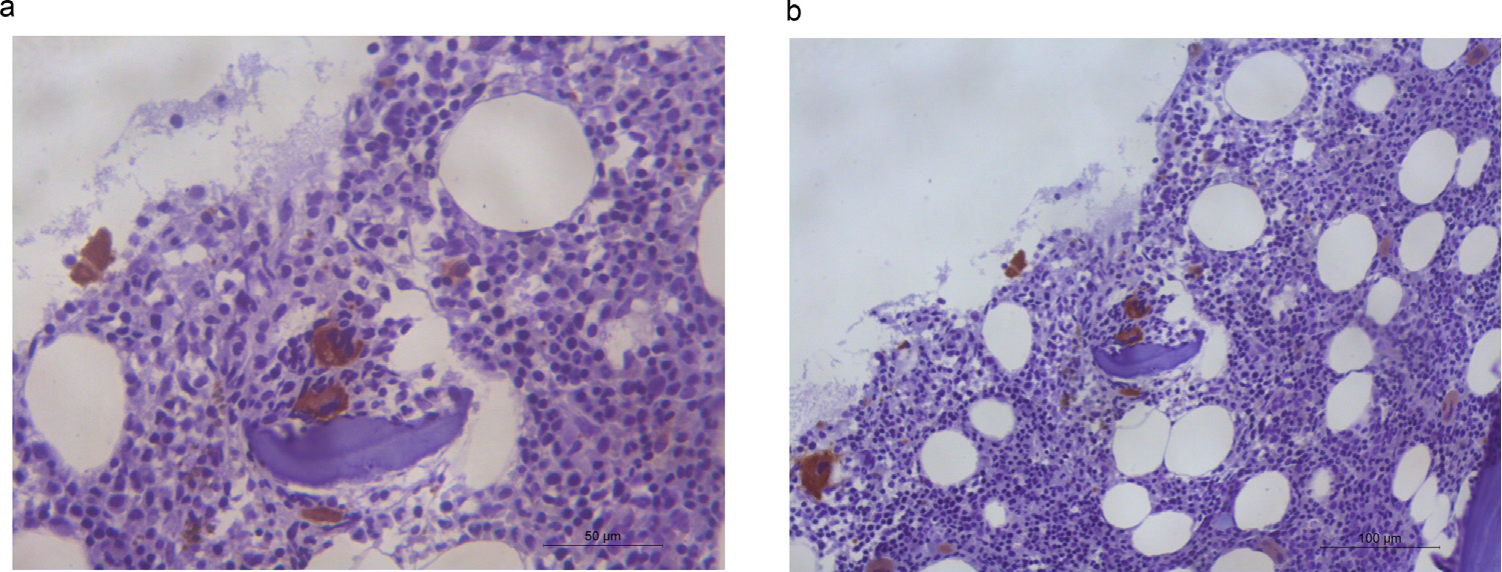
Immunohistochemical analysis of CD61 antibody in bone marrow aspirate smear showed a dysplastic megakaryocyte in an unusual peritrabecular area (a. ×20 and b. ×40).

In healthy adult subjects, megakaryocytes are usually found, individually, adjacent to the bone marrow sinusoid and away from bone trabeculae. Peritrabecular dysplastic megakaryocytes are extremely rare and this abnormal architectural finding, beyond cell dysplasia, corroborates the diagnosis of MDS.^1^

Morphological dysplasia is still of paramount importance in the diagnosis of MDS, even in the molecular genetic era. The presence of at least one cytopenia is required for diagnosis. Bone marrow aspiration examination with iron stain, biopsy and cytogenetics testing standard karyotypes are all considered mandatory. Genetic testing for somatic mutations in genes associated with MDS is highly recommended, although not universally available. Therefore, conventional diagnostic tools and risk prognostic models will continue to have a role in the clinical practice.^1^^,^^2^

The case we present emphasizes an unusual morphologic abnormality. Accurate diagnoses have implications for both decision-making and prognosis.

## Author contributions

MMSR, FDRF, RFP and SMMM: Conceptualization, Visualization, Laboratorial analysis, Writing—original draft, and writing—review and editing. HLRJ and PSM: Visualization, Writing—original draft, and Writing—review and editing.

## Conflicts of interest

The authors have no conflicts of interest to declare.
